# Long term follow up of high risk children: who, why and how?

**DOI:** 10.1186/1471-2431-14-279

**Published:** 2014-11-17

**Authors:** Lex W Doyle, Peter J Anderson, Malcolm Battin, Jennifer R Bowen, Nisha Brown, Catherine Callanan, Catherine Campbell, Samantha Chandler, Jeanie Cheong, Brian Darlow, Peter G Davis, Tony DePaoli, Noel French, Andy McPhee, Shusannah Morris, Michael O’Callaghan, Ingrid Rieger, Gehan Roberts, Alicia J Spittle, Dieter Wolke, Lianne J Woodward

**Affiliations:** Department of Obstetrics and Gynaecology, The University of Melbourne, Melbourne, Victoria Australia; Department of Paediatrics, The University of Melbourne, Melbourne, Victoria Australia; Victorian Infant Brain Studies, Clinical Sciences, Murdoch Childrens Research Institute, Parkville, Victoria Australia; Premature Infant Follow-up Program, The Royal Women’s Hospital, Parkville, Victoria Australia; National Women’s Hospital, Auckland City Hospital, Auckland, New Zealand; Department of Neonatology, Royal North Shore Hospital, Sydney, New South Wales Australia; Newborn Services, The Royal Women’s Hospital, Parkville, Victoria Australia; Department of Paediatrics, University of Otago, Christchurch, New Zealand; Department of Paediatrics, Royal Hobart Hospital, Hobart, Tasmania Australia; Neonatal Paediatrics, King Edward Memorial Hospital, Perth, Western Australia Australia; Neonatal Services, Women’s and Children’s Health Network, North Adelaide, South Australia Australia; Paediatrics and Child Health, Mater Clinical School, University of Queensland, Brisbane, Queensland Australia; Department of Neonatal Medicine, Royal Prince Alfred Hospital, University of Sydney, Sydney, New South Wales Australia; Faculty of Medicine, University of Sydney, Sydney, New South Wales Australia; Community Child Health, The Royal Children’s Hospital, Parkville, Victoria Australia; Department of Physiotherapy, The University of Melbourne, Parkville, Victoria Australia; Department of Psychology, The University of Warwick, Coventry, UK; Department of Pediatric Newborn Medicine, Brigham and Women’s Hospital, Harvard Medical School, Boston, USA; Department of Obstetrics and Gynaecology, The Royal Women’s Hospital, 20 Flemington Road, Parkville, Victoria 3052 Australia

**Keywords:** Infant, Low birth weight, Preterm, High-risk, Follow-up, Cognition, Development, Growth

## Abstract

**Background:**

Most babies are born healthy and grow and develop normally through childhood. There are, however, clearly identifiable high-risk groups of survivors, such as those born preterm or with ill-health, who are destined to have higher than expected rates of health or developmental problems, and for whom more structured and specialised follow-up programs are warranted.

**Discussion:**

This paper presents the results of a two-day workshop held in Melbourne, Australia, to discuss neonatal populations in need of more structured follow-up and why, in addition to how, such a follow-up programme might be structured. Issues discussed included the ages of follow-up, and the personnel and assessment tools that might be required. Challenges for translating results into both clinical practice and research were identified. Further issues covered included information sharing, best practice for families and research gaps.

**Summary:**

A substantial minority of high-risk children has long-term medical, developmental and psychological adverse outcomes and will consume extensive health and education services as they grow older. Early intervention to prevent adverse outcomes and the effective integration of services once problems are identified may reduce the prevalence and severity of certain outcomes, and will contribute to an efficient and effective use of health resources. The shared long-term goal for families and professionals is to work toward ensuring that high risk children maximise their potential and become productive and valued members of society.

**Electronic supplementary material:**

The online version of this article (doi:10.1186/1471-2431-14-279) contains supplementary material, which is available to authorized users.

## Background

There are now approximately 300,000 babies born every year in Australia, most of who survive the newborn period, and then grow and develop normally. However, clearly identifiable groups of survivors, such as those born preterm or with ill-health, have adverse long-term outcomes, with higher than expected rates of health or developmental problems, compared with children born at term and in good health.

Usual health surveillance for the majority of children comprises regular visits to a Maternal and Child Health Nurse (or equivalent), or to a primary care doctor for checks on the child’s health, growth and development, and to organise immunisations. Follow-up rates for these services are high during infancy and steadily fall during the preschool years. Those who are at higher risk are worthy of a more structured and specialised programme of follow-up assessments, for at least two reasons. First, families want to know if their child is healthy and growing and developing normally, or if health or other problems are likely to be encountered in the future. Special concerns often arise at transition points, such as entering child care or changing school levels, thus requiring careful guidance and advice. Second, some problems can be ameliorated or prevented if detected early – identification of high-risk groups for targeted intervention can be both effective and efficient.

The number of studies reporting long-term outcomes of high-risk children is rapidly increasing. However, the methods of these follow-up studies vary widely, with sources of variability including inclusion criteria, timing of assessments, outcome measures and use of appropriate control groups. As a result, it is difficult to compare the results of published studies, or to aggregate the data to provide more certainty around the rates of some important outcomes. Outcome statistics are needed for counseling families as well as to guide practitioners who follow high risk groups to know what outcomes can be expected and at what ages specific problems may emerge. This information can also be linked to evidence-based pathways to assist families to access management resources.

While it is widely accepted that high-risk infants need close monitoring and surveillance, a framework for this practice has only infrequently been developed; for example in one instance for a selected group of infants over the first 5 years of life [1]. In addition to being useful for informing clinical follow-up programs, a framework could also assist longitudinal research with high-risk infants and help to improve uniformity of follow-up time-points and measures. The aim of this report is to provide a framework for identifying which children need specialised follow-up, what outcomes should or could be of interest, in addition to how, where and when follow-up should be conducted.

## Main text

A two-day workshop was held in Melbourne, Australia, on March 24 and 25, 2011. Invited health professionals representing paediatrics, psychology, nursing, occupational therapy, and physiotherapy, as well as parents of high-risk children attended the workshop. The workshop panel included participants from across Australia and New Zealand, as well as an eminent researcher from the United Kingdom. This paper is a summary of discussions from that workshop, with further modifications in the months following.

### Who needs follow-up?

Children may require specialised follow-up for reasons specific either to the child or their family. Risk factors are likely to be additive, with increased risk of adverse outcomes as the number of risk factors increases.

#### Child variables

The following children are at higher risk for long-term health problems, particularly neurosensory impairments:

- Preterm (PT; gestational age <37 weeks); higher risk – very preterm (VPT; <32 weeks); highest risk - extremely preterm (EPT; <28 weeks), or
- Low birth weight (LBW; birth weight <2500 g); higher risk – very low birth weight (VLBW; <1500 g); highest risk – extremely low birth weight (ELBW; <1000 g).
- Small for gestational age (SGA – <3^rd^ percentile or < −2 SD weight for gestational age and sex) infants are also at higher risk, but would also usually fall into one of the LBW categories.
- Neonatal encephalopathy (including seizures), regardless of cause
- Term babies who have received positive pressure ventilation for >24 hours
- Congenital brain or heart malformations, genetic syndromes or inborn errors of metabolism that affect neurodevelopmental outcomes
- Failed newborn hearing screening
- Neonatal central nervous system infections – meningitis/encephalitis
- Infants requiring major surgery (e.g., brain, cardiac, other thoracic or abdominal)
- Hyperbilirubinaemia (bilirubin >400 μmol/l or clinical evidence of bilirubin encephalopathy)
- Neurobehavioural abnormalities noted in the newborn period.

#### Family/environmental variables

- High social risk (e.g., domestic violence, previous child abuse, severe poverty or homelessness)
- Substance abuse by either parent
- Major psychiatric history in either parent
- Developmental disability in either parent.

### Why do they need follow-up?

The reasons for needing follow-up can be considered from the perspectives of the child/parents/family, ethics, and audit/research.

#### Child/parental/family viewpoint

- To provide a clinical service for families – there is an onus on those who provide neonatal care to high-risk babies to ensure that the baby’s care beyond the nursery is optimised. Although this does not mean necessarily that neonatal health care providers must provide follow-up services, each high-risk family should have a clear follow-up pathway mapped out on discharge from the nursery. In an ideal world every identified child would be assigned a case worker who is responsible for case management, at least until school entry.
- Parents perceive a lack of long-term outcome information about their high-risk children. Such information is also needed for other health professionals, including family or allied health practitioners, and the education system.
- To provide accurate information that might influence decisions about initiation of intensive care or redirection to palliative care.
- To provide accurate information to families at the optimal time to facilitate decision-making for important life events (e.g. school choices, deferred or delayed school entry), screening and assessment for developmental disorders (e.g. Autism Spectrum Disorder), and monitoring for less visible medical conditions (e.g. hypertension).
- To provide advocacy for families – identify the need for assessment and treatment resources, and then seek funding from appropriate sources to ensure these resources are adequate and provided when they are needed and most effective.
- To provide a resource for schools and educators about the specific problems of the at-risk groups (e.g. very preterm) and how best to remediate these.

#### Ethical viewpoint

- ‘Intensive care’ does not cease on discharge from the nursery. Neonatal intensive care units (NICUs) need to appreciate the ongoing morbidities for infants that survive and are discharged home. This is especially useful to help identify improvements in both the quality of treatment and outcomes of care, as well as to be able to identify those at highest risk for monitoring and early intervention.
- To provide ongoing support to vulnerable children and their families.
- To provide data on long-term outcomes to future carers or service providers for the child and family.
- To ensure that high-risk children’s and their families’ best interests are paramount in the face of concerns about the high costs of providing neonatal intensive care.

#### Audit/research

- To fill gaps in knowledge, and hence to understand short and long term outcomes more precisely, especially long-term post-discharge outcomes.
- To establish the burden of illness from certain clinical groupings.
- To provide data for benchmarking, which can be fed back to service providers, not only to those involved in perinatal (obstetric or paediatric) care, but also beyond, including to providers of intervention and early education services, the school system and later health providers, including behavioural health and psychiatric services.
- To determine the consequences of treatments or other interventions or exposures, not only from randomised controlled trials, but also other study designs, such as cohort studies designed to monitor long-term effects of new treatments that are implemented into clinical care. Such treatments have not always been evaluated first in randomised controlled trials, and even if they have, ongoing surveillance is warranted to detect rare or long-term adverse effects that cannot be detected in the initial trials.
- To establish a framework for follow up of at-risk children, including identifying targets and best timing for intervention.
- To identify causal pathways, and in particular risk and resilience factors.
- To generate hypotheses for further research, such as future randomised controlled trials of interventions or exposures designed to improve long-term outcomes for high-risk children and their families.

### How should high-risk children be followed?

Several issues need to be addressed as part of this question. These include consideration of the important outcomes of interest, when children should be assessed, the personnel required, and what psychometrically sound, assessment tools are available.

#### What outcomes are of interest?

The outcomes to be assessed vary with the child’s age (Additional file 1: Table S1). The emphasis is to identify relevant areas, from both the child’s and the family’s perspective, and how these might vary in importance at particular ages.

#### Child variables

Child variables can be grouped into four broad domains: physical health, mental health, learning and cognition, and quality of life. Dependent upon the resources and time available, the number of domains assessed at each time point could vary from few, such as in a busy general practice setting, to most in a well-resourced research setting, provided the outcomes were helpful in answering the clinical or research questions being proposed, and in supporting the children and their families. The method of assessment may also vary depending on the setting, from screening questionnaires, or office-based clinical assessment, to referral to specialised services such as educational psychology or cardiology.

#### Physical health

Physical health can include a number of different areas, as follows:

i. General health
  Regardless of the age of the child, any current or ongoing health problems must always be addressed, particularly because they could affect performance in other areas of the child’s development. Some of these health problems could result from complications arising in the newborn period, such as ongoing breathing difficulties and oxygen dependency from lung injury.
ii. Growth
  Height, weight and head circumference will be relevant at all ages, but particularly in early life when growth rates are high and when aberrations of growth, particularly poor growth, detected by plotting the measurements on growth charts relevant for the age and sex of the child, can be indicators of underlying health problems. After the first few years growth in all measurements slows, apart from during puberty, and poor growth is less likely to present as a new problem. Overweight and obesity, on the other hand, are ongoing health problems throughout life, and are increasingly common in recent years in both childhood [2] and adulthood [3] in Australia and elsewhere.
  Other measurements, such as mid-arm circumference or skin-fold thickness might be relevant for some research questions, but are not usually helpful in clinical practice. The waist-to-hip ratio currently is valuable for emphasising life course cardiovascular preventative health [4].
iii. Feeding problems

Some babies might feed poorly, including some with oral aversions, before discharge home. Others with either ongoing health problems or evolving neurological problems might have feeding problems, sometimes requiring prolonged tube feeding after discharge or later in life. Management of these underlying problems may lead to food refusal and poor growth [5]. Support for lactating mothers, including those who are breast feeding their babies, should be available, including referrals to lactation consultants or speech pathologists, as required.

iv. Special senses
  Major visual and hearing problems are often diagnosed before discharge home as eye examinations are routine for some high-risk groups, such as those born VPT or VLBW, although criteria may vary according to local experience. Hearing screening is universal for most babies in Australia prior to discharge home, and in many other regions of the world. Other high-risk groups who may not have had such assessments before discharge might need specialised follow-up by ophthalmologists or audiologists. Other children identified to have abnormal visual or hearing function should be referred for assessment. In later childhood, more subtle optical problems and visual processing disorders occur more frequently in some high-risk groups [6], and these subtle problems can interfere with learning. Similarly, hearing disorders other than deafness, such as short-term auditory memory problems or figure-ground perceptual problems (difficulty hearing in a noisy background) are more prevalent in some high-risk groups, and can interfere with learning, particularly in the classroom [7].
v. Neurological
  As neurological problems, especially cerebral palsy, are much more prevalent in high-risk children, neurological assessment is particularly important in the early years. Severe cerebral palsy will present earlier in childhood, usually within the first year after birth, with disordered tone and tendon reflexes, along with abnormal motor development. Milder cerebral palsy, however, may not be diagnosed for certain until later, sometimes after the child has started to walk. A recent systematic review describes the prevalence, type and distribution of cerebral palsy according to gestational age [8]. Any degree of cerebral palsy should be graded according to the Gross Motor Function Classification System (GMFCS) [9].
vi. Motor skills
  Apart from cerebral palsy, many high-risk children have delayed motor development during infancy. Monitoring motor milestones is important as some children with initial motor delay will catch up, whilst others will have ongoing poor motor function or incoordination, and some may later be diagnosed with Developmental Co-ordination Disorder (DCD) [10]. Referral to a physiotherapist for a standardised motor assessment may be required in the presence of significant motor delay, or to an occupational therapist for fine-motor delays that affect activities of daily living, such as hand-writing. The pooled prevalence of motor impairment in high-risk preterm children without cerebral palsy is 19% for moderate impairment and 40% for mild-moderate impairment [10].
vii. Cardiovascular health
  Reliable blood pressure measurements are difficult to obtain in the first few years of life in an ambulatory setting. Although it is easy enough to obtain a reading in younger children, particularly with an oscillometric device, rarely can a reliable resting blood pressure be obtained prior to school-age. Blood pressure measurements later in childhood are important in some high-risk groups; those born EPT or ELBW have higher blood pressure than controls [11], and hypertension is an important precursor to adult cardiovascular disease.
  Assessments of cardiac function, such as echocardiography or pulse wave velocity, are predominantly research tools, in the absence of clinical symptoms or signs of cardiovascular disease.
viii. Respiratory health
  Some babies are discharged home with ongoing respiratory problems, which might, for example, involve home oxygen therapy. Other children fall into higher-risk groups for recurrent respiratory illnesses, some of whom might require readmission to hospital. Respiratory illnesses comprise one of the major reasons for admission to hospital in the first few years after discharge home in both preterm and term children [12]. Some high-risk children have higher rates of airway hyper-reactivity, sometimes manifesting as asthma. It is important to monitor respiratory exacerbations in asthmatic children and the impact of airway hyperreactivity on sleep and school attendance.
  Respiratory function tests are possible on infants and young children, but are of limited utility beyond the research setting. By school-age, children are able to co-operate with standard respiratory function measures, but again respiratory function testing is mostly a research tool, in the absence of respiratory symptoms or signs.
ix. Metabolic/endocrine
  Tests for metabolic or endocrine diseases would not be part of a normal assessment battery in the absence of symptoms or signs of disease. However, such tests might be appropriate for some research questions.
  Metabolic bone disease is more common in some infants, such as those born very tiny or preterm [13, 14], but rarely presents clinically in childhood, apart from during the newborn period. Some children might be at higher risk of the Metabolic Syndrome (diabetes mellitus, hyperlipidemia and cardiovascular disease) in adult life [14].
x. Reproduction
  Reproductive issues, either preventing pregnancy or trying to achieve pregnancy will assume increasing importance in later life and into adulthood.

#### Learning and cognition

i. Cognitive development
  Regardless of the age of the child a major part of any assessment, whether for clinical or research purposes, will involve an evaluation of a child’s cognitive development.
  Cognitive development varies widely, and not all children develop specific skills at the same rate. There are numerous methods for assessing cognitive development more formally in early childhood, which will be discussed later. In infancy and toddlerhood assessment tools tend to focus on detecting those children who are delayed, and in preschoolers and beyond assessment tools are more specific and focus on identifying strengths and vulnerabilities across different cognitive domains. As children approach school age, it is usually possible to move from tests of global development to more in-depth assessments of cognitive function, including measures of intelligence quotient (IQ), executive function, memory and attention.
ii. Language
  Adequate language development is fundamental to communication and normal social interaction. Language development is complex, beginning from birth and evolving rapidly. In early life children’s language skills are typically divided into expressive (what the child says) and receptive skills (what the child understands), with children typically understanding more than they can say in the early years. As they grow older, language abilities become more differentiated – a recent systematic review of language functioning summarises the various aspects of language and the results of language studies from very preterm or very low birth weight children [15]. In some extremely preterm children voice abnormalities can occur post-intubation, and be associated with poor self-esteem, if not proactively managed [16].
iii. Pre-academic skills
  As children approach school-age it is appropriate to ask if they are ready for school and whether additional support might be warranted to support this important life transition. An assessment of school readiness should include consideration of a child’s health and physical development, emotional well-being and self-regulation, social competence, approaches to learning, communication skills, and cognitive ability. As can be seen, these cut across other areas listed in this section. However, there is merit, both clinically and from a research viewpoint, in undertaking a developmental check-up of a child’s strengths and vulnerabilities to enable the early identification of those at high risk of later learning problems who would benefit from closer educational surveillance and/or proactive support. Depending on the resources available, educational referral for very preterm children with problems in either one or multiple school readiness domains may be warranted [17]. In addition to assessing the child’s developmental needs it will also be important as part of this check-up to consider wider ecological factors such as the readiness of the school for the child and the extent of family/community psychosocial adversity since these factors will also affect a child’s educational potential.
iv. Academic progress
  At school age, numeracy and literacy should be assessed at regular intervals to ensure adequate progress in these areas [18]. Some countries, such as Australia, have introduced standardised testing of academic skills at several points during the school years. In children with significant cognitive or learning disorders, individualised educational programming is essential so that they can achieve their ‘personal best’ rather than constantly comparing their progress with their peers.

#### Mental health

Mental health variables will alter with the age of the child, as follows:

i. Behaviour
  Some high-risk infants demonstrate altered behaviour from birth, and even more by term equivalent age, if born preterm. This is particularly so for infants who are born very preterm, have a neurological abnormality, have high medical needs or have had an extensive period of hospitalisation. Appreciation of the complexity of neonatal behaviours has developed remarkably; a comprehensive neurobehavioural evaluation will help to increase our understanding of an infant’s behaviour, including their strengths and vulnerabilities, enabling care and parent education to be adjusted accordingly. These examinations also assist to identify those most at risk of developmental disabilities, enabling further assessment and intervention as early as possible [19]. Whist in the neonatal nursery, babies’ neurobehavioural development is often evaluated and developmental interventions provided, continuation of such programs is less common in the community.
  In the first weeks after discharge home infant behaviour revolves around sleeping, which babies do most of the time, and crying. Infants with altered self-regulation can exhibit considerable problems with sleeping and settling, and how they interact with their family. Some babies demonstrate intense or difficult temperaments from a very early age [20]. Crying patterns vary over the first year of life [21]. As wakefulness increases with age, the child may confront stage-specific behavioural challenges, and different patterns of behaviour emerge, some of which may persist over time [22], or can evolve into various regulatory and behavioural disorders, as outlined later [23].
ii. Social skills
  In early infancy babies begin to learn how to interact with their environment and start to become more alert and responsive, including early social communication through smiling, eye contact and vocalisation. Differences in social abilities compared with peers may begin to manifest later in infancy and may be a marker of an underlying developmental disorder. Social skills are crucial for integration in the peer group. Children who differ in individual characteristics that may range from physical differences (e.g. small stature) or psychological traits (e.g. shyness, poor attention, lower cognitive abilities) that are more often found in at risk children, are at increased risk of being socially excluded and bullied [24]. Being bullied has been shown to have a range of highly adverse long term consequences and needs to be addressed early [25].
iii. Daily functioning
  Later in the first year and into second year of life children want to take more responsibility in their own care, for example they may want to feed themselves. Toileting, dressing and other self-care issues, such as getting around the neighbourhood, become important later in childhood.
iv. Other behavioural health disorders
  Towards the end of the first year and into the second year, some infants may demonstrate signs of social withdrawal and delayed language development, which might be an early indication of the presence of Autism Spectrum Disorder.
  Later in life other psychopathologies, such as Attention Deficit-Hyperactivity Disorder, and other externalising behaviours evolve, along with mood and anxiety, and other internalising disorders. In the teenage years and later a small minority may be at risk for major psychiatric disorders [26].

#### Quality of Life

i. Daily functioning
  Skills in daily living, also known as adaptive skills, such as with feeding, dressing, toileting, communication, mobility, socialisation and emotional regulation, appropriate for the age of the child, are all important to consider.
ii. Self-esteem and well-being
  Aspects of quality of life include the child’s feeling of well-being or self-esteem, life satisfaction, function within society, including within peer groups, and the ability to form and maintain relationships. From mid-to-late-childhood, young people can, and should, self-report on these measures, and should be explicitly counselled that they can set goals and achieve home, educational and community success.

### Family variables

#### Parents’ mental health

At all ages, but particularly soon after birth and discharge home, assessment of parental mental health, especially of the primary caregiver to the child, is important, as problems are most prevalent soon after birth. For children to be nurtured optimally parental mental well-being is vital. Moreover, for children with special needs, parental mental health and well-being could vary over time.

#### Carer-child interaction

Even with adequate parental mental health, parental ability to provide a nurturing environment can vary, depending on past experience and family history [27]. Problematic parent–child interactions might be ameliorated by providing parents with skills to help them understand their baby’s needs and behaviour, as well as interact with their child in a way that helps scaffold child learning and foster successful social behaviour.

#### Social support

Social isolation and stressful life events can interfere with growth and development, even if carers do not have a clearly defined mental health disorder.

#### Siblings

The effect of siblings on a new child’s development can vary widely, as can the effect on the siblings of the arrival a new member of the household. Sibling relationships can be supportive but if problematic they can also have highly detrimental effects on mental health [28].

### When should high-risk children be assessed?

- Routine follow-up assessments should be more frequent immediately after discharge, with the interval between assessments extending as the child grows older.
- Follow-up for specific ongoing or new health problems will influence the timing of some assessments, over and above routine follow-up assessments.

Suggested ages for follow-up assessments are listed in Additional file 1: Table S1. It should be noted that for preterm children, especially those who are very preterm (<32 weeks) or extremely preterm (<28 weeks) ages should be adjusted for the degree of prematurity if the primary goal of follow-up is for research, because there is a bias in cognitive test scores if they are not corrected for prematurity that is largest in early childhood, but also persists into school-age [29, 30]. However, the bias introduced by not correcting for prematurity may lead to the child scoring below a threshold that makes them eligible for additional resources, and hence correcting for prematurity might be counterproductive if the goal of the assessment is to obtain additional help for the child, particularly in the pre-school or early school years. Other than for research, correction for prematurity is not helpful beyond the first few years, particularly if the child is being compared with similar age peers. Scores obtained for research purposes that have been adjusted for prematurity could be re-scored using the date of birth to determine if a child qualifies for assistance within the education system.

### Who should be involved in the assessments?

The personnel involved will be dependent on the number of areas to be assessed. Under most current circumstances in clinical practice there will a primary care provider, such as a general practitioner or a Maternal and Child Health Nurse, who should refer to specialists for assessment and management when indicated. In a research setting, however, there may be many other health professionals, such as cardiologists, ophthalmologists, audiologists, respiratory physicians, psychologists, speech pathologists, nurses, physiotherapists or occupational therapists, depending on the areas that need to be assessed. Ideally in clinical practice, children with multiple areas of concern, or risk, should be referred for assessment by a multidisciplinary team of health professionals.

### What assessment tools can help?

The methods and assessment tools selected will depend on the expertise of the examiner, the time and resources available, and the presenting concerns. In the section below, we suggest examples of available tools, but acknowledge that many other options are also available. Assessors should use tools with which they are familiar and that yield results that are locally relevant. For example, a Maternal and Child Health Nurse may screen development using a parent completed screener [31, 32] but then refer to a psychologist or other suitable health professional, such as a physiotherapist or developmental paediatrician, for a detailed standardised developmental assessment, if concerned.

### Physical health

i. General health is best assessed by standard history taking and physical examination, with an emphasis on the neurological system; subsequent assessments would be guided by the responses elicited.
ii. Growth – standard anthropometric measurement of weight, height (length &lt;2 years of age) and head circumference with accurate measuring devices; the child’s size for these measurements can be compared with other children of the same age and sex using the WHO Child Growth Standards, available at http://www.who.int/childgrowth/standards/en/index.htm Waist-to-hip ratio, mid-arm circumference and skin-fold thickness measurements may be relevant in some circumstances.
iii. Feeding problems can be assessed using a parent-completed questionnaire [5].
iv. Special senses – assessment of visual and auditory functioning might be required for some high-risk children or in those with visual or hearing problems at any assessment.
v. Neurological examination – with particular emphasis on motor function, tone and tendon reflexes.
vi. Motor skills – the properties of various infant motor scales have been recently reviewed [33], including Prechtl’s General Movements Assessments [34], the Alberta Infant Motor Scale (AIMS) [35], the Test of Infant Motor Performance (TIMP) [36], the Neuro-Sensory Motor Development Assessment (NSMDA) [37] and the motor scales of Bayley Scales of Infant and Toddler Development [38]. Abnormalities of Prechtl’s General Movements Assessments in the first months of life, particularly the absence of normal fidgety movements by 3 months of age, can be predictive of the later development of cerebral palsy in high-risk children, whilst the TIMP is able to identify motor delay from 32 weeks’ gestation up until 4 months’ corrected age [39]. The AIMS, NSMDA and Bayley can be used from 1 month but are less useful at the bottom end of their validated age ranges, and are better from 4 months onwards. Whilst these tools can be useful in identifying children at risk of motor impairments, including cerebral palsy, false positives and negatives are common and ongoing follow-up is recommended [40, 41]. In older children standardised motor assessments are applicable if there is a concern about motor performance. From three years of age fine and gross motor function can be assessed with the Movement Assessment Battery for Children-Second Edition [42], or the Bruininks-Oseretsky Test of Motor Proficiency, Second Edition (BOT-2), either screener or full version [43].
vii. Cardiovascular health – assessments might include either clinical sphygmomanometry, using a standard sphygmomanometer or an oscillometric device, or could involve 24-hour ambulatory blood pressure monitoring. Other cardiovascular investigations, usually in a tertiary or research setting, could include echocardiography for assessing ventricular function, or measurements of arterial intima-media thickness, pulse wave velocity or vascular reactivity.
viii. Respiratory health – symptoms can be assessed with International Study of Asthma and Allergies in Childhood (ISAAC) questionnaire [44], and respiratory function testing might involve spirometry, measurement of lung volumes or exercise tolerance in suitable children who can comply with instructions. In addition, the 6-minute walk test could be considered as a summary assessment of cardiorespiratory function [45].
ix. Metabolic/endocrine – tests for metabolic bone disease, carbohydrate intolerance, and lipid profile might be indicated in some children, particularly those who are overweight or obese.
x. Reproduction – in older children or adults specific tests for fertility, both male and female, might be indicated.

### Learning and cognition

i. General development – The Bayley Scales of Infant and Toddler Development – Third Edition [38] are widely used to assess developmental progress over the first 3½ years. The Griffiths Mental Development Scales-extended/revised can also be used from birth up to 8 years of age, but at the older end of the test it is really only useful for children with delayed development.
ii. From about 2½ years of age cognitive functioning can be assessed by a variety of IQ tests [46]. The Wechsler scales [47, 48] are the most commonly used battery for the assessment of general intellectual functioning and are often the basis for integration assistance within the education system. Other batteries that provide reliable estimates of general cognitive functioning include the Stanford-Binet Intelligence Scales, Differential Ability Scales, and Kaufman Assessment Battery for Children.

While tests of general intellectual function provide valuable information, they are not sensitive to deficits in specific cognitive domains. Specific neuropsychological tests are required in order to identify the nature of attention, memory, executive function and information processing deficits. While numerous neuropsychological measures are available, well validated tests to consider are the NEPSY-II [49] which assesses a range of domains from 3 to 16 years, the Test of Everyday Attention for Children (TEACh) [50] for the assessment of attention from 6 to 15 years, the Children’s Memory Scale [51] for the assessment of memory and learning skills from 5 to 16 years, the Automated Working Memory Assessment [52] for the assessment of working memory, and the Delis-Kaplan Executive Function System [53] and the Behavior Rating Inventory of Executive Function [54] for the assessment of executive functioning.

iii. Language development - There are several measures available for the assessment of language development. Early language development can be assessed with the Rosetti Infant-Toddler Language Scale from birth to three years of age [55], the MacArthur-Bates Communicative Development Inventories (CDI-II: 8 to 37 months) [56], the Preschool Language Scale (PLS4: birth to 6 years) [57], and the Clinical Evaluation of Language Fundamentals – Preschool (CELF-P2: 3 to 6 years) [58]. For school-aged children validated measures for the assessment of speech and language skills include the Clinical Evaluation of Language Fundamentals – Fourth Edition (CELF® - 4) [59], the Test of Language Competence – Expanded Edition (TLC-Expanded: 5 to 18 years) [60], and the Comprehensive Assessment of Spoken Language (CASL: 3 to 21 years) [61].
iv. Pre-academic skills – Consider subtests from the Pre-school Screening Test [62] which aims to identify children who will require additional resources when they transition to school. It is designed for children aged 3:6 to 4:5 years, and the screening test takes 10–15 minutes. Alternative measures include the Early Math Diagnostic Assessment [63] for screening children at risk of difficulties with mathematics from pre-kindergarten, the Early Reading Diagnostic Assessment (ERDA-II) [64] for screening children at risk of reading difficulties from kindergarten, and the Process Assessment of the Learner (PAL-II) [65] for screening reading and writing problems from kindergarten.
v. Academic skills at school age – The Wechsler Individual Achievement Test (WIAT) [66] is Australian standardised and provides a comprehensive assessment of academic abilities. There is also an abbreviated version (WIAT-II Abbreviated) which can assess reading, spelling and numerical ability in 10–20 minutes. An alternative is the Wide Range Achievement Test (WRAT4) [67], which assesses word reading, sentence comprehension, spelling and mathematics. When standardised assessments are not available, teacher reports in the short Teacher Assessment of Academic Skills provide a highly reliable measure [68].

### Mental health

Behaviour, or neurobehaviour, in the newborn period and early months can be evaluated using a number of standardised assessments, such as the NICU Network Neurobehavioural Scale [69] and the Einstein Neonatal Neurobehavioral Assessment Scale [70]. The appropriateness of the scale will depend on the infant’s age at assessment and the examiner’s experience and training. A recent review of neurobehavioural evaluations may assist clinicians and researchers in their selection of assessments [19]. Later behavioural outcomes can be assessed by a large range of questionnaires. The Infant-Toddler Social and Emotional Assessment [71] is a comprehensive parent-report measure of social, emotional and behavioural problems and competencies in children aged 12 to 36 months. Well known instruments such as the Child Behavior Checklist [72], and the Behavioral Assessment System for Children (BASC-2) [73] have preschool and child versions. Alternatives to these well standardised and expensive measures include the Tester’s Rating of Child Behavior [74], and the Strengths and Difficulties Questionnaire [75], which is brief, can be freely downloaded, and is applied in many clinical and research settings due to its strong psychometric properties. Using both parent and teacher reports enhances diagnostic accuracy of behaviour disorders [76].

i. Mental health diagnoses, such as anxiety, depression, other internalising disorders, conduct disorder, major psychiatric illnesses (especially psychosis), autism, attention-deficit/hyperactivity disorder (ADHD), or obsessive compulsive disorder can be assessed with the Preschool Age Psychiatric Assessment (PAPA: 2 to 5 years) [77], the Development and Well-Being Assessment (DAWBA: 5–17 years) [78], the Diagnostic Interview for Children and Adolescents (DICA-IV) [79], the Children’s Interview for Psychiatric Syndromes (ChIPS: 6 to 18 years) [80], and the Structured Clinical Interview for DSM Disorders (SCID: adults) [81].
ii. Autism screeners – The Modified Checklist for Autism in Toddlers [82] is useful in screening for autism between 16 and 30 months, although Maternal and Child Health Nurses can also be trained to refer children with signs of autism as young as 12 months of age [83]. For older children autism screeners include the Gilliam Autism Rating Scale (GARS-2), the Social Communication Questionnaire (SCQ: 4 years +) and the Social Responsiveness Scale (SRS: 4 to 18 years).
iii. ADHD screeners – The Brown Attention Deficit Disorder Scales for Children and Adolescents (3 to 18 years), and Conners 3^rd^ Edition (Conners 3™; 6–18 years) [84] are useful in screening for ADHD.
iv. Risk-taking behaviour in adolescents can be assessed with HEADSS framework, which provides a structure for an interview about the young person’s Home, Employment and education, daily Activities, Drug taking, Sexuality, and Suicide risk [85].

### Quality of life

i. Daily functioning skills can be assessed using parent or teacher questionnaires such as the Vineland Adaptive Behavior Scales [86], which can be applied from birth through to adulthood to assess communication, daily living skills, socialisation, and motor skills.
ii. Well-being and self-esteem can be either self-reported if the child is old enough to understand the questions, or parent-reported in younger children. The aim is to establish the child’s well-being, life satisfaction, function within society/peer group, and ability to form and maintain relationships. Some of these aspects can be captured by assessments such as the Health Utility Index Mark 3 [87]. Self-esteem can be assessed by questionnaires such as the Coopersmith Self-Esteem Inventory [88].

### Family variables

i. Mental health of the parents can be assessed by a range of questionnaires assessing mood and anxiety, such as the Center for Epidemiologic Studies Depression Scale [89], the Depression Anxiety Stress Scales [90], the Beck Depression Inventory [91], the Beck Anxiety Inventory [92] and the Hospital Anxiety and Depression Scale [93].
ii. Parent–child interaction and relationships can be assessed by a range of tools depending on the age of the child [94]. The interaction session may be unstructured, semi-structured or structured (e.g., Etch-a-Sketch [95]), and various coding systems are available, some of which require extensive training and are designed for use by trained specialists.
iii. Family function can be assessed by enquiring about social circumstances of the family, such as social support, education and employment of the primary caregiver, and maternal age, and computing a social risk index [96], or by using standard questionnaires such as the Family Assessment Device [97] or the Parenting Stress Index [98].
iv. Siblings’ health and functioning can be assessed by any of the above methods, depending upon the areas of concern.

### What should be reported?

The detail of what is reported will vary depending on who needs the information and why. Clearly reports need to be written in a way that allows families to understand the results and which give clear guidance for management. Although reports for health professionals might include highly technical detail, this should be presented in a way that is accessible to all readers.

Whatever is reported and regardless of the target of the report, the assessment should summarise strengths, as well as weaknesses that require support, of both the child and the family.

### What are the challenges in translation into practice?

#### Clinical care

If the major goal of follow-up is to provide ongoing clinical care for a high-risk child, a minimal checklist of areas to cover for a health professional, such as a paediatrician, would be represented by the shaded areas in Additional file 1: Table S1. Depending on what is found at each assessment, referral for more thorough evaluation of some areas might be required. If progress is otherwise satisfactory, then follow-up could be limited to the areas highlighted, but occasionally other issues might require assessment.

The issue of how long to follow a child who is progressing normally and whose family is coping appropriately will depend on many circumstances. Children at two years who are walking freely with no abnormal neurological signs, talking in 2–3 word sentences, with no vision or hearing problems, whose parents have no major behavioural concerns, and who have no other health problems that require ongoing care, and whose family is functioning well, can be discharged from specialist surveillance and referred back to primary care services, with a clear management and follow-up plan. Whether later assessments at preschool or early school ages are warranted could be decided on an individual basis and informed by screening during primary care visits or by family concerns. However, it must be recognised that many of the cognitive and academic problems that occur at school-age will not be predicted by progress in the first few years of life. Many high-risk children will not be able to function adequately in the preschool or school setting. They will remain undiagnosed and unassessed until they have failed and been brought to the attention of someone at an age that is usually too late for effective intervention. Ideally the highest risk children should be reassessed prior to school entry, and after the first few years at school. The reality is that resources are limited, and few centres are able to offer such assessments routinely, even though the cost of follow-up is minimal compared with the cost of providing intensive care. While the cost of follow-up needs to be acknowledged, the cost of inaction, which is more difficult to measure, may be much greater. Moreover, one of the other difficulties experienced by families is that once they are ‘out of the system’, it is hard to get back in to mobilise the resources required quickly. As a minimum, parents of high risk children, health professionals and educators need to be “alert but not alarmed” and advised that serious issues can still arise at later times. Maintaining good records of previous assessments will facilitate reassessments at later ages.

### Research

#### Age and outcomes assessed

If the major reason for following children is for research, then the ages at which the child is assessed and the outcomes being assessed will be determined by the research questions being addressed. It is not possible to assess all outcomes on all children and their families at each time point, or to assess the children indefinitely. The costs of follow-up and the need to find the money to answer the research questions will ultimately limit the number of assessments and the outcomes being determined. In addition, exhaustive assessments can be exhausting for the child and family, and run the risk of reducing compliance with all aspects of the assessment from tiredness or lack of concentration, as well as reducing the likelihood of co-operation with future assessments, if they are planned.

#### A suggested minimum data set for research

For those contemplating follow-up for research purposes a minimum data set that might be able to be compared more directly with other studies would include anthropometric measurements, neurological function (diagnosis of cerebral palsy and its severity using the GMFCS [9]), cognitive function (developmental quotient or IQ), vision (legal blindness, or visual acuity in both eyes) and hearing (deafness requiring hearing aids or worse, or decibel loss in each ear).

#### Control groups in research

In addition to correction of cognitive test scores for prematurity, discussed earlier, a comparison group is critical in research studies, as cognitive tests normed on one population at one point in time do not necessarily translate well across countries and across time [99]. With an appropriate control group, if results of cognitive tests are reported as mean, SD and number of subjects for both the high-risk group and the controls, it is possible to compute standardised mean differences between groups and their confidence intervals, to allow for comparison between groups, as well as for pooling of the data with other studies.

Within randomised controlled trials the use of a control group is obvious. For a cohort study of high-risk individuals, the challenge is to obtain a comparison group who are low or zero risk for the exposure of interest (such as extremely preterm birth or low birth weight), but of otherwise equal risk on social variables and other possible confounders, such as sex of the child. Control groups can be recruited contemporaneously with the high-risk group, for example from birth, or can be recruited at later ages from the general population. Both methods have their strengths and weaknesses. Recruitment from birth allows more accurate recording of obstetrical and neonatal data, but there is the problem of attrition over time, with those who drop-out potentially changing the baseline risk, as well as reducing the power of the study as the sample size reduces. Recruitment at later ages from the community allows one to obtain sufficient numbers of controls, but ensuring an appropriate balance for confounders can be difficult, particularly as those who volunteer for such studies may have different risk from the general population.

#### Duration of follow-up

Concerning the duration of follow-up for research purposes, there is a compromise between assessing children at early ages (to obtain immediate data for feedback to clinicians and families and ensuring high follow-up rates), and assessing children later in life (allowing more detailed assessments, but running the risk of loss to follow-up and biased results, as those who are easier to follow-up have lower rates of problems than those who are followed with more difficulty) [100]. It must be remembered that developmental outcomes measured during infancy and early childhood are moderate predictors of long-term development, particularly for scores on cognitive tests [101].

### Improved information sharing and integrated service provision

Increasingly, web-based information platforms such as the Raising Children Network (http://raisingchildren.net.au/newborns/newborns.html) are allowing families to connect more easily with other parents and professionals. A widely used electronic health record, if sufficient uptake can be achieved, may be a way forward to seamlessly share health information with relevant stakeholders.

### Best practice for families

For the families of children who have either been born very preterm or who have had a stay in the intensive care nursery after birth for other reasons, the current services on offer after discharge vary enormously from region to region and from hospital to hospital. As the people who will know their children better than anyone else, it will fall to the families to be aware of the likely problems that their child might encounter and to be an advocate for the needs of their child. Parents should receive information about follow-up support available after discharge and individual counselling about the likely long-term problems for their child, along with written information to reinforce the messages. The timing and content of information provided to parents will depend on the follow-up services available. Appropriate websites should also be given so that the parents do not have to search among the multitude of advice on the World Wide Web, much of which may be inappropriate for their circumstances.

### What are the research gaps in long-term outcomes?

- The child.

- The concept of school readiness needs to be clarified and defined – it is a complex construct with child-specific physical, social, emotional, and cognitive components, as well consideration of whether the school is ready for the child, and broader factors such as the adequacy of family and community supports [102].
- Much of the research has focused on risks rather than factors that protect at risk children from adverse outcome or contribute to resilience (e.g. such as the family). More resiliency research is required.
- More early interventions, before, during or after birth, as well as after discharge, must be developed to reduce the excessive rates of adverse long-term outcomes for high-risk children. There have been some successes in improving long-term outcomes from trials of magnesium sulphate before very preterm birth [103], caffeine therapy after birth for infants <1250 g birthweight [104], and early interventions after discharge [105], but more are needed. Outcomes from any trials of early intervention should be designed to target specific outcomes, and the outcomes need to encompass more than just developmental or intellectual functioning of the child. Such outcomes could include not only specific health problems, such as neurological, respiratory or cardiovascular health, but also quality of life of individuals and their families, and other behavioural, social and functional outcomes. Importantly, interventions that may be helpful at school age and not just in infancy are needed and are promising new avenues to be explored [106, 107].
- More standardisation of the types of high-risk subjects, their ages and the outcomes assessed would facilitate the pooling of data, and the ability to bench-mark outcomes between centres and regions.

b) The parents.

Parental mental health is relevant to outcomes for all children, but particularly for high-risk children. Moreover, parental mental health is more evident in those with high-risk children than controls [108].

- Parental mental health is an under-researched area, particularly in fathers.
- The evolution of mental health symptomatology in mothers, from pre-pregnancy until well beyond infancy.
- Mental health problems need to be diagnosed more by clinical interview, rather than relying solely on questionnaires.
- The risk factors for parental mental health problems need to be identified, and ameliorated.
- Translation into practice
- Genetic and epigenetic changes related to the causes and consequences of high-risk infants are not currently translatable into clinical practice, but may prove useful in detecting higher-risk individuals in the future.
- Results of any research into improved outcomes for high-risk children need to be widely accessible and applied quickly to clinical practice. Web-based resources and parent support groups in partnership with academic and clinical experts will help to facilitate these processes.

## Conclusions

Preterm and high-risk births are common: the vast majority of these neonates are discharged from the nursery into community care. This is an important public health and economic issue: a substantial minority of these children has long-term medical, developmental and psychological adverse outcomes that consume extensive health and education services. Early intervention to prevent adverse outcomes and the effective integration of required services as soon as problems are identified may reduce the prevalence or severity of certain outcomes, and will contribute to an effective and efficient use of health resources over the child’s life course.

Traditionally, research has focused on identifying long-term difficulties and assessing the efficacy of interventions in the NICU and in early childhood.

We are now in a position to:

○ More clearly identify research gaps

○ Address the gaps in translation and communication of these results

○ Make clear recommendations about how best to follow-up these at-risk children

○ Recommend that funding for neonatal intensive care should include a commitment to fund follow-up assessments for high-risk children, at least until early school-age.

The shared long-term goal for families and professionals is to work toward ensuring that high risk children maximise their potential and become productive and valued members of society.

## Electronic supplementary material

Additional file 1: Table S1: Child and family outcomes to be considered at different ages. (DOCX 20 KB)

## Abbreviations

ADHD: Attention-deficit/hyperactivity disorder
BASC: Behavioral assessment system for children
CASL: Comprehensive assessment of spoken language
CDI: Communicative development inventories
CELF – 4: Clinical evaluation of language fundamentals – fourth edition
CELF-P2: Clinical evaluation of language fundamentals – preschool
DAWBA: Development and well-being assessment
DCD: Developmental coordination disorder
DICA: Diagnostic interview for children and adolescents
DSM V: Diagnostic and statistical manual of mental disorders, fifth edition
ELBW: Extremely low birth weight
EPT: Extremely preterm
ERDA: Early reading diagnostic assessment
GARS: Gilliam autism rating scale
GMFCS: Gross motor function classification system
HEADSS: Home, employment and education, daily activities, drug taking, sexuality, and suicide
ICD: International classification of diseases
IQ: Intelligence quotient
LBW: Low birth weight
NICU: Neonatal intensive care unit
PAL: Process assessment of the learner
PAPA: 2 to 5 years: Preschool age psychiatric assessment
PLS4: Preschool language scale
PT: Preterm
SCID: Structured clinical interview for DSM disorders
SCQ: Social communication questionnaire
SGA: Small for gestational age
SRS: Social responsiveness scale
TEACh: Test of everyday attention for children
TLC-Expanded: Test of language competence – expanded edition
VLBW: Very low birth weight
VPT: Very preterm
WIAT: Wechsler individual achievement test
WRAT: Wide range achievement test.
