# Supplementary material for: Long term follow up of high risk children: who, why and how?
Source: BMC Pediatr. 2014 Nov 17;14:279. doi: 10.1186/1471-2431-14-279 (PMC4289257; doi:10.1186/1471-2431-14-279)
Supplement: Supplementary file 1 — Additional file 1: Table S1: Child and family outcomes to be considered at different ages. (DOCX 20 KB) [file 12887_2014_1205_MOESM1_ESM.docx]

**Table 1** Child and family outcomes to be considered at different ages

|  | **Ages at assessment** | | | | | | | | | | | |
| --- | --- | --- | --- | --- | --- | --- | --- | --- | --- | --- | --- | --- |
|  | 2-6 w | 3-4 m | 8 m | 12 m | 15-18 m | 24 m | 36 m | 4-5 y* | 6-8 y† | 12-14 y | Transition to adult | Adult |
| **Child** |  |  |  |  |  |  |  |  |  |  |  |  |
| **Physical Health** |  |  |  |  |  |  |  |  |  |  |  |  |
| general health | +++ | +++ | +++ | +++ | +++ | +++ | +++ | +++ | +++ | +++ | +++ | +++ |
| growth | +++ | +++ | +++ | +++ | ++ | ++ | ++ | ++ | ++ | +++‡ | ++§ | ++§ |
| feeding problems | +++ | ++ | ++ | ++ | + | + | + | 0 | 0 | 0 | 0 | 0 |
| special senses | +++ | ++ | ++ | + | + | + | + | + | + | + | + | + |
| neurological | +++ | +++ | +++ | +++ | +++ | +++ | ++ | ++ | + | + | + | + |
| motor skills | + | ++ | ++ | +++ | +++ | +++ | +++ | +++ | +++ | ++ | + | + |
| blood pressure/CVS | UR | UR | UR | UR | UR | +/- | +/- | ++ | +++ | +++ | +++ | +++ |
| respiratory health | +++ | +++ | +++ | +++ | +++ | +++ | ++ | ++ | +++ | +++ | +++ | +++ |
| metabolic/endocrine | 0 | 0 | 0 | 0 | 0 | 0 | 0 | 0 | + | ++ | +++ | +++ |
| reproduction | 0 | 0 | 0 | 0 | 0 | 0 | 0 | 0 | 0 | + | ++ | +++ |
| **Learning and cognition** | |  |  |  |  |  |  |  |  |  |  |  |
| Development/ cognitive function | ++ | ++ | ++ | ++ | +++ | +++ | +++ | +++ | +++ | +++ | ++ | ++ |
| language | + | ++ | +++^ | +++^ | +++^ | +++^ | +++ | +++ | +++ | + | 0 | 0 |
| pre-academic skills | 0 | 0 | 0 | 0 | 0 | 0 | + | +++ | ++ | 0 | 0 | 0 |
| academic progress | 0 | 0 | 0 | 0 | 0 | 0 | 0 | 0 | +++ | +++ | +++ | ++¶ |
| **Mental Health** |  |  |  |  |  |  |  |  |  |  |  |  |
| behaviour | +++ | +++ | +++ | +++ | +++ | +++ | +++ | +++ | +++ | +++ | +++ | +++ |
| social skills | + | + | ++^ | +++^ | +++^ | +++^ | +++^ | +++ | +++ | +++ | +++ | +++ |
| psychopathology | 0 | 0 | 0 | +^ | +^ | ++^ | ++^ | ++ | +++ | +++ | +++ | +++ |
| risk-taking behaviour | 0 | 0 | 0 | 0 | 0 | 0 | 0 | 0 | 0 | ++ | +++ | +++ |
| **Quality of Life** |  |  |  |  |  |  |  |  |  |  |  |  |
| daily functioning | ++ | ++ | ++ | ++ | ++ | ++ | +++ | +++ | +++ | +++ | +++ | +++ |
| quality of life | 0 | 0 | 0 | 0 | 0 | 0 | + | ++ | +++ | +++ | +++ | +++ |
| **Family** |  |  |  |  |  |  |  |  |  |  |  |  |
| parents' mental health | +++ | +++ | +++ | +++ | +++ | +++ | +++ | +++ | +++ | +++ | +++ | +++ |
| carer-child interaction | +++ | +++ | +++ | +++ | +++ | +++ | +++ | ++ | + | + | + | 0 |
| family function | +++ | +++ | +++ | +++ | +++ | +++ | +++ | +++ | +++ | +++ | +++ | +++ |
| siblings | +++ | +++ | +++ | +++ | +++ | +++ | +++ | +++ | +++ | +++ | +++ | +++ |

0 = does not apply; + to +++ reflects relative importance; +/− = of dubious value.

w = weeks; m = months; y = years; CVS = cardiovascular system; UR = unreliable.

*prior to school entry; †1-2 years after starting school; ‡growth 12–14 years includes normal pubertal development; §overweight/obesity an ongoing issue; ¶ongoing life learning; ^relevant to early presentation of autism spectrum disorder.

Shaded areas represent a suggested minimal checklist for busy clinicians.
